# Autophagy, cancer and angiogenesis: where is the link?

**DOI:** 10.1186/s13578-019-0327-6

**Published:** 2019-08-13

**Authors:** Bahareh Kardideh, Zahra Samimi, Fatemeh Norooznezhad, Sarah Kiani, Kamran Mansouri

**Affiliations:** 1 Immunology Department, Faculty of Medicine, Kermanshah University of Medical Sciences, Kermanshah, Iran; 2 Medical Biology Research Center, Health Technology Institute, Kermanshah University of Medical Sciences, Kermanshah, 6714967346 Iran; 3 Molecular Medicine Department, Faculty of Medicine, Kermanshah University of Medical Sciences, Kermanshah, Iran

**Keywords:** Autophagy, Vessel cell biology, Angiogenesis

## Abstract

**Background:**

Autophagy is a catabolic process for degradation of intracellular components. Damaged proteins and organelles are engulfed in double-membrane vesicles ultimately fused with lysosomes. These vesicles, known as phagophores, develop to form autophagosomes. Encapsulated components are degraded after autophagosomes and lysosomes are fused. Autophagy clears denatured proteins and damaged organelles to produce macromolecules further reused by cells. This process is vital to cell homeostasis under both physiologic and pathologic conditions.

**Main body:**

While the role of autophagy in cancer is quite controversial, the majority of studies introduce it as an anti-tumorigenesis mechanism. There are evidences confirming this role of autophagy in cancer. Mutations and monoallelic deletions have been demonstrated in autophagy-related genes correlating with cancer promotion. Another pathway through which autophagy suppresses tumorigenesis is cell cycle. On the other hand, under hypoxia and starvation condition, tumors use angiogenesis to provide nutrients. Also, autophagy flux is highlighted in vessel cell biology and vasoactive substances secretion from endothelial cells. The matrix proteoglycans such as Decorin and Perlecan could also interfere with angiogenesis and autophagy signaling pathway in endothelial cells (ECs). It seems that the connection between autophagy and angiogenesis in the tumor microenvironment is very important in determining the fate of cancer cells.

**Conclusion:**

Matrix glycoproteins can regulate autophagy and angiogenesis linkage in tumor microenvironment. Also, finding details of how autophagy and angiogenesis correlate in cancer will help adopt more effective therapeutic approaches.

## Background

Autophagy is a mechanism of degrading intracellular components. The subunits produced after autophagy could be reused by the cells. In this process, long-lived proteins, damaged organelles, and other cytoplasmic components are encapsulated in double membrane vesicles to form autophagosomes. These autophagosomes would be further integrated with lysosomes and their contents would be degraded by lysosomal enzymes [1]. Based on the size of the substrate and degradation rate, autophagy is classified into four classes: macroautophagy, microautophagy, chaperone-mediated autophagy, and selective autophagy. Different types of autophagy have common characteristics for degrading lysosomal proteins; but through different mechanisms [2]. Macroautophagy is a biologic process conserved from yeast to human that eliminates long-lived proteins and damaged organelles through which components are trapped in double-membrane vesicles called autophagosomes. Although the origin of this membrane is dubious, intracellular organelles such as endoplasmic reticulum (ER), mitochondria, and others could be the source [1, 3]. Nutrients deficiency, hypoxia and oxidative stress are the main triggers for macroautophagy [2]. Although the triggers of micro-autophagy are known poorly up until now, it has been established that the soluble constituents are translocated into lysosomes directly. These cytoplasmic substances are invaginated in an endosomal/lysosomal membrane and form vesicles in lysosomes degraded by lysosomal enzymes [4]. Chaperone-mediated autophagy (CMA), on the other hand, is stimulated by starvation, oxidative stress, and factors that could damage proteins. During CMA, substrate proteins are targeted to the lysosomal membrane by chaperone molecules such as Hsp70 and subsequently translocated to the lysosomal lumen to be further degraded [4]. The CMA substrate proteins have specific amino acidic motif recognized by Hsp70. Although this process is not well-understood, it facilitates targeted proteins translocation to lysosome lumen [5]. In selective autophagy, the specific cargos are marked with ubiquitin and targeted for degradation in autophagosomes. So, in this type of autophagy, the cargos such as misfolded proteins and damaged organelles are selected for degradation. Based on the cargo, the selective autophagy has been named, for example, mitophagy for degradation of mitochondria, lipophagy for degradation of lipids and pexophagy for degradation of peroxisome [2, 6].

**Fig. 1:**
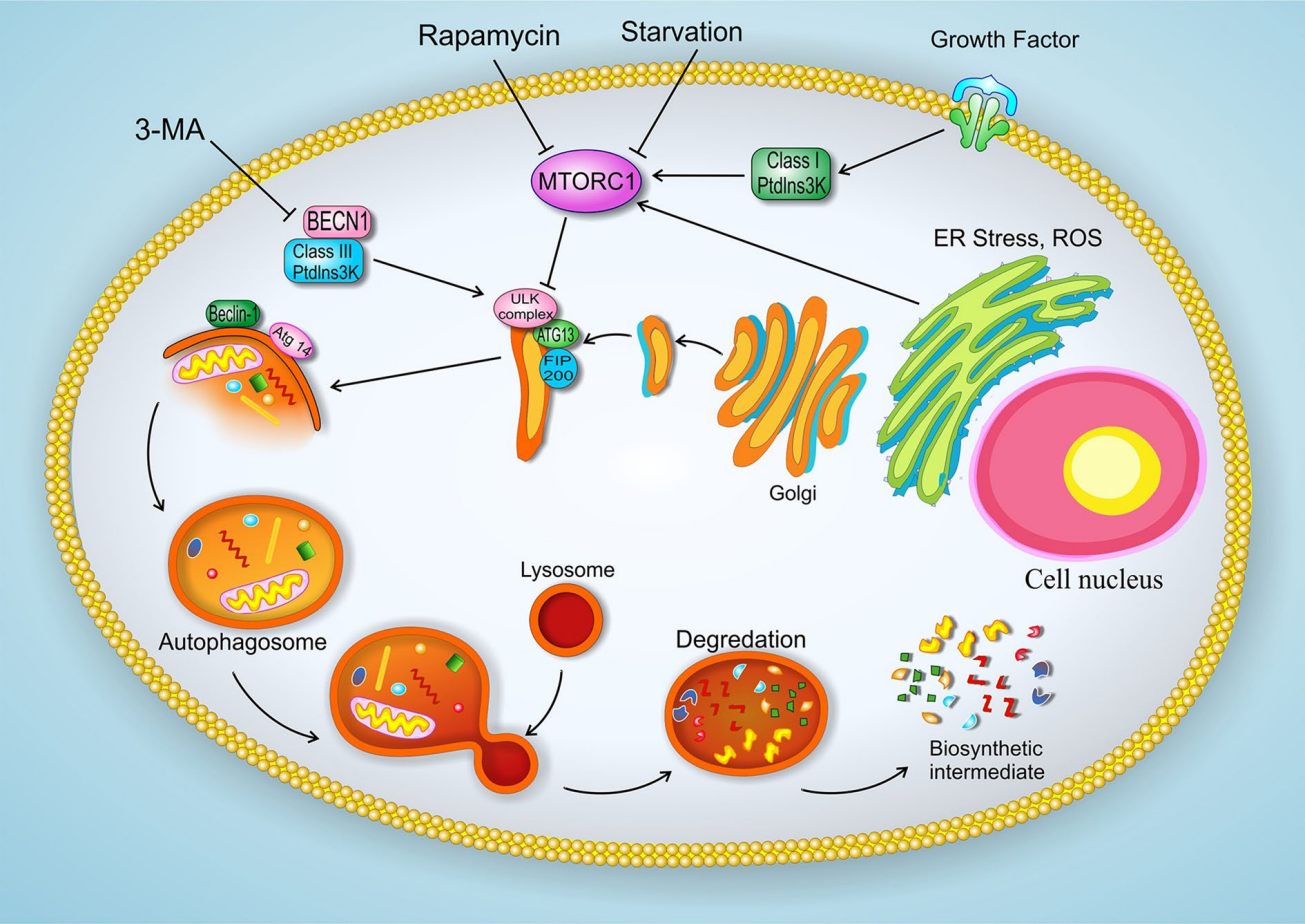
The autophagy pathways in endothelial cells. Autophagy pathway consists of several steps including induction, autophagosomes nucleation, elongation, fusion with lysosomes, and degradation of autophagosomes contents

Autophagy in mammals was studied from 1960. Research on autophagy in yeast has been assisting our understanding of its molecular mechanism; since the autophagy-related proteins were first known in yeast. These proteins homologous were discovered in human and other mammalian models later [7, 8]. Autophagy pathway consists of several steps including induction, autophagosomes nucleation, elongation, fusion with lysosomes, and degradation of autophagosomes contents (Fig. 1) [9]. Atgs proteins are necessary for autophagosome formation and autophagic cargo delivery to lysosomes. The Unc-51-like kinase 1 (ULK1) complex is essential for the initiation step. It consists of ULK1, Atg13 and FIP200. Beclin-1 and Atg14 are most involved molecules in the nucleation of autophagosome. Two conjugated complexes of Atg12-Atg5-Atg16L1 and phosphatidylethanolamine-microtubule associated light chain 3 (PE-LC3) have critical importance in phagophores expansion. Although the detailed mechanism of autophagosome and lysosome fusion are poorly characterized, the molecules such as SNAREs, syntaxin 17 (STX17) on the autophagosome and vesicle associated membrane protein 8 (VAMP8), on the lysosome have been known to mediate [10]. Autophagy, through its conserved mechanism, can breakdown macromolecules and provide a pool of nutrients; so, it has a critical role in cell homeostasis. Autophagy defect can affect the intracellular conditions and associate with different diseases such as aging, neurodegenerative disease, lysosomal disorders, and cancer [11]. Although autophagy has a controversial role in cancer, some studies have considered it as a tumor suppressor mechanism. Deletion or defect in many of these autophagy-related genes (Atgs) has been reported to associate with tumor progression [12, 13]. Furthermore, autophagy is important to keeping the endothelial cell (EC) homeostasis under both normal and pathological conditions [14, 15]. According to the fact that angiogenesis plays a critical role in tumor progression, this review tends to focus on the link between autophagy and angiogenesis in cancer. Comprehending the relation between angiogenesis and autophagy in cancer would present more exact strategies for combinatorial therapy.

## Autophagy and cancer

The role of autophagy in cancer is rather twisted. The evidence shows that the suppression of Atgs accelerates tumor promotion while the expression of autophagy proteins is declined in many cancer cell types [12, 16]. Accordingly, autophagy could also present an anti-tumor feature. On the other hand, autophagy maintains the intracellular homeostasis and recycles the nutrients for cells; so, it could promote tumorigenesis [17].

### Autophagy and tumor suppression

The Atgs expression levels have been shown to significantly decline or be totally lost in many cancer cell types. So, it is proposed that autophagy has anti-tumor characteristics [18]. The evidence supporting this claim could be classified by different aspects. In certain cancer cells, the Atgs have been shown to carry mutations and monoallelic deletions. For instance, in many cases of breast, prostate and ovarian tumors, the Bcl-2-interacting myosin-like coiled-coil *(Beclin*-*1*) gene, an autophagy-related gene, hasa monoallelic deletion [19, 20]. Also, in a large number of brain cancers, the expression of *Beclin*-*1* is significantly decreased [21, 22]. Besides mutations and deletions, the expression level of Atgs declines in special types of cancer cells. In this regards, it is worth mentioning the framework mutations in Atg2B, Atg5, Atg9, and Atg12 which could promote the risk of gastric and colorectal cancers development [18]. Regarding the role of these genes, we can say that Atg2 is an essential gene for autophagosomes formation in yeast [18]. Although the precise role of Atg9 remains to be determined, this membrane protein has been shown to contribute in phagophore maturation in eukaryotes cells [23]. As mentioned in the previous section, Atg5 and Atg12 are associated with Atg16L1 to form a conjugation system necessary for expansion of phagophores [24]. Furthermore, Bif-1 is a member of Endophilin protein family with two domains of N-BAR and Src homology 3 (SH3). It can regulate Atg9 vesicle conformation during autophagy. In the cells with depleted Bif-1 pool, the budding of membrane and Atg9 vesicle trafficking are reduced in response to autophagy inducers such as starvation [25]. Also, the expression level of Bif-1has been shown to be significantly reduced in colon adenocarcinoma [26]. Therefore, it could be clearly concluded that any deletion or decrease in the level of expression of Atgs can positively influence cancer promotion; which in turn signifies the anti-tumorigenesis features of autophagy. Interestingly enough, upon a decline in the expression of Atgs in normal cells, the rate of tumor formation would increase gradually [17]. For example, in the Atg6 ± mice, the rate of tumorigenesis at old ages is higher compared to wild types [27]. Interestingly, autophagy could play both paradoxical roles of tumor suppression and tumor promotion in cancer. The tumor microenvironment is the factor determining which way this double-edged sword will go. Primarily, autophagy is considered as a tumor suppressor mechanism due to its importance in intracellular balance maintenance. This function of autophagy on the other hand, is combined with its role in cell death and immune-surveillance and eventually tumor inhibition [28]. When the autophagy level is subdued, the cells lose the ability to eliminate toxic agents such as long-lived proteins and damaged organelles. These cells would face the accumulation of p62/SQSTM1 (p62), reactive oxygen species (ROS), and other detrimental materials [29]. Interrupted autophagy can lead to structural and functional defects due to the instability of the intracellular environment. These cells usually carry more damaged DNA molecules and uncontrolled proliferation in response to metabolic stress compared to the cells with normal autophagy level. Based on this evidence, reduced autophagy can increase tumorigenesis susceptibility [17, 29]. On the other hand, when autophagy is disrupted, the damaged organelles and aggregated proteins are accumulated in the cells and cause cellular deformity [30]. Another pathway through which autophagy suppresses tumorigenesis is cell cycle regulation. Autophagy in cooperation with ubiquitin–proteasome system (UPS) can modulate cell cycle progression and cell death through some key cell cycle regulators of CDK-Cyclin complexes [31]. The mammalian target of Rapamycin (mTOR) is an important negative regulator of autophagy with a critical role in cell proliferation induction through receptor tyrosine kinases (RTKs) [25]. Although other autophagy-related proteins can inhibit cell proliferation, their exact mechanism is not precisely determined yet. For instance, a monoallelic deletion in *Beclin*-*1* gene in immortalized epithelial cells can raise their growth rate [32]. Furthermore, deletion in one copy of UV radiation resistance-associated gene (UVRAG), a regulator of beclin-1/PI3 K, promotes human colon cancer by inhibiting autophagy. It could be concluded that reduced autophagy associates with tumor progression by increasing cancer cell proliferation [17, 33].

**Fig. 2:**
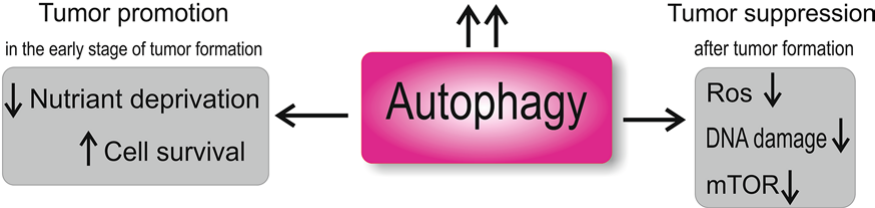
The effect of Autophagy on regulation of tumorigenesis. In the early stages of tumor formation, autophagy facilitates cell survival by overcoming nutrient deprivation; but after tumor formation, it oppositely suppresses tumor promotion through mTOR and ROS regulation

Autophagy is also an important regulator of proliferation in cells with apoptosis defects; as upon inefficient apoptosis, the cancer cells gain the opportunity to escape programmed cell death. Under this circumstance, autophagy plays a determinant role in the fate of these cells (see Fig. 2). Before angiogenesis occurs in solid tumors, cancer cells go through lack of nutrients and oxygen. As a result, cancer cells with defected autophagy would go through long-term chronic necrosis [10]. Moreover, inflammatory cells such as cytotoxic T cells and NK cells migrate towards this site and invade the tumor cells. This in turn enhances cell proliferation and angiogenesis. These immune cells may also need autophagy for activation; since in antigen presenting cells, autophagy is important to antigen presentation to T lymphocytes. Therefore, autophagy can suppress tumorigenesis through activating immune cell [34].

### Autophagy and tumor promotion

Autophagy has a dual role in tumorigenesis regulation. Despite the fact that autophagy is known as a tumor suppressor mechanism, there are studies indicating that it could also enhance tumors promotion. For example, an elevated level of autophagy has been demonstrated in cancer cells going through metabolic stress and facing reduced nutrient supply. During the early stages of tumor formation, cancer cells endure nutrients limitation and hypoxia due to tumor growth and reduced blood supply which in turn decrease cancer cell proliferation and keep them in a steady state [35]. In solid tumors such as breast cancer and melanoma, increased level of LC3 is correlated with aggressiveness of these cancers [36]. In addition, in cancer stem cells (CSCs), autophagy maintains intracellular balance. For example, an increased level of autophagy is needed for breast cancer and leukemia development [37, 38]. In other words, cancer cells use autophagy as a survival mechanism and resume their proliferation upon improvement in the condition.

### Autophagy and vascular biology

There is numerous evidence on the role of autophagy in normal vascular biology. Autophagy can regulate the endothelial cells (ECs) homeostasis through several mechanisms. One of the important routes is regulating redox homeostasis. Although oxidative stress on a low degree could improve ECs function; it could also promote angiogenesis by these cells in high levels of ROS [39]. Autophagy is crucial for regulating redox state in ECs which exact mechanism is not determined yet. Nonetheless, autophagy preserves cytoplasmic and bioenergetics balance through removal of damaged organelles and reducing ROS production [40]. For example, a previous study has shown that certain compounds such as curcumin could induce autophagy in ECs rendering protective effects on cardiovascular cells [41]. In another study by Wang et al. it was found that autophagy in endothelial cells could be increased by ROS generation. This elevated autophagy can maintain cell survival under stress condition [42]. Furthermore, autophagy can maintain ECs homeostasis by affecting lipid metabolism. A recent study has shown that impaired autophagy in ECs can increase atherosclerotic plaque development under high stress conditions. Also, adequate autophagy in ECs can limit atherosclerotic plaque formation through inhibiting apoptosis, senescence and inflammation [43]. On the other hand, lipid accumulation in atherosclerotic lesions indicates that autophagy can preserve the normal vascular function through targeting lipid droplets, also known as lipophagy [44]. Another mechanism through which autophagy affects angiogenic factors in normal condition is regulating receptors such as VEGFR2. The ECs protect the VEGFR2 from cleavage by endocytosis [45]. When the ECs are in the quiescent state, the turnover of VEGFR2 is reduced as a supporting function for resting condition. It seems that autophagy interferes with the availability of VEGFR2 by regulating endocytic pathway [46, 47]. Moreover, inadequate autophagy could be involved in vascular aging and pathological complications. In endothelial and smooth muscle cells, the triggers like oxidative lipids and β-amyloid promote autophagosome formation. Furthermore, autophagy has a critical role in some vascular events such as calcification and angiogenesis [15]. Based on previous studies, autophagy can regulate nitric oxide (NO) bioavailability and down-regulate ROS and inflammatory cytokines production [48, 49]. Recent studies claim that autophagy flux can limit the destructive effects of angiotensin II on ECs. Furthermore, the increase of angiotensin II and oxidative stress during mellitus diabetes could lead to vascular damage. Under such circumstance, autophagy is vital to ECs since it protects them through inhibition of glucose-induced vascular stress [50]. This data clearly suggests a regulatory role of autophagy under both normal and stress conditions in vascular endothelial cells. This level of regulation is achieved through enhancing the homeostasis and establishment of an inflammatory and anti-inflammatory balance [48].

### Autophagy in vascular endothelium of tumors

Autophagy has a dual role in various stages of tumor development. Prior to tumor formation, autophagy plays a suppressive role; it can maintain cytoplasmic balance through degradation of cytotoxic compartments and limiting inflammation [51]. After tumor formation, autophagy acts differently and increases the nutrients supply for tumors; so, it contributes to cancer cells survival [52]. Tumor microenvironment (TME) contains extracellular matrix and certain stromal cells including fibroblasts, immune cells and endothelial cells (ECs). These cells are originated from both surrounding tissues and bone marrow. However, autophagy effects regarding these stromal cells are not exactly determined yet. Despite the importance of autophagy in crosstalk between cancer cells and stromal cells, it contributes to vascular compartment to finally play a pivotal role in determining the fate of cancer cells [47]. The ECs in TME face nutrient deprivation, hypoxia, low glucose and low blood flow. This condition changes vessel function and activate vascular sprouting. The tumors vessel structure is different from normal vasculogenesis. For instance, tumors vessels show high permeability, low stability and different diameters in comparison to a normal vessel [53]. These weak structural features lead to disrupted blood flow and limit oxygen and nutrients accessibility. In this stressful situation, the ECs use autophagy to escape from these stressors [54]. The study by Ou X and coworkers indicated that under oxidative stress, heightened autophagy mediated by SIRT-1 can increase ECs survival. SIRT-1, an NAD-dependent deacetylase affects autophagy through PI3K/Beclin-1 and m-TOR pathways [55]. On the other hand, the vulnerable integrity in tumors vessels is due to perturbation in adherence and tight junctions between ECs and other surrounding cells. For example, the declined expression of endothelial Cadherins (such as CDH5) can increase vascular permeability [47, 56]. Maes et al. showed in their study that Chloroquine (CQ), known as an autophagy inhibitor, can improve tight junctions between ECs and other stromal cells. So, inhibition of lysosomal degradation by CQ can decrease metastasis and tumor invasion [57]. Also, Schaafs study demonstrates that CQ can confine phosphorylation of VEGFR2 by VEGFA in ECs. Having in mind the fact that the interaction between VEGFR2 and VEGFA is the key mechanism of angiogenesis, it seems that CQ decreases vascular sprouting in TME [58]. Furthermore, increased autophagy in Tumor-associated ECs (TECs) supports their access to nutrients under metabolic stressors like hypoxia. In addition to boosting autophagy in these cells, hypoxia can induce angiogenesis by stabilizing α-subunite of Hypoxia-Inducible Factor (HIF)-complex and intervening with VEGF signaling [54]. In the next section, we would explain more about the relation between autophagy and angiogenesis, focusing on matrix proteoglycans.

### Autophagy and angiogenesis

One of the less studied conditions to which autophagy becomes so vital is the link between autophagy and angiogenesis. Angiogenesis is a process during which new capillaries are formed from previous vessels. It occurs in many physiological processes such as organ growth, wound healing and also in pathological situations like tumor growth and metastasis [59, 60]. Tumor angiogenesis occurs mainly by nearby ECs and differs from vessel construction from bone marrow originated endothelial precursor cells (vasculogenesis). The balance between pro- and anti-angiogenic factors is a regulator of tumor angiogenesis. ECs in tumor site are exposed to high levels of VEGF, nutrient deprivation and hypoxia; all being vessel sprouting inducers [47]. Under hypoxia, cells start releasing the angiogenic factors such as VEGF. The ECs are activated by receiving the signals of these factors through their corresponding receptors. Matrix metalloproteinases are also released from activated ECs and facilitate the process of angiogenesis. Other molecules like integrins associate with the growth of new blood vessels [61]. Eventually, the newly formed vessels are stabilized by pericytes and smooth muscle cells surrounding these structures. Recent studies indicate that hypoxia induces angiogenesis and promotes cancer cell metastasis through hypoxia-inducible factor (HIF). Under normoxia, HIF-1α is hydroxylated and subsequently degraded. HIF-1α, on the other hand, is stable under the hypoxic condition and could be translocated to the nucleus to enhance the angiogenic factors expression [62, 63]. The ECs in tumor microenvironment can balance out the stress by increasing autophagy flux [47]. A recent study shows that Atg5 depletion in ECs can exacerbate functional abnormality in tumor vasculature. This could indicate the importance of autophagy for ECs homeostasis [57]. The research regarding the relation between autophagy and angiogenesis renders inconsistent results. For example, Rapamycin-induced autophagy mediates pro-angiogenic effects in the HUVECs. Based on this study, autophagy can enhance angiogenesis by affecting AMPK/Akt/mTOR signaling [64]. Also, increased production of Vascular Endothelial Growth factor A (VEGF-A) in the ischemic myocardium model of acute myocardial infarction (AMI) in mice can induce angiogenesis through ROS-ER stress-autophagy pathway in vascular endothelial cells [65]. On the other hand, another research illustrates that Mebendazole (MBL) associates with anti-angiogenic effect in ECs through autophagy induction which could be a novel target for cancer treatment [66].

Interestingly enough, the extracellular matrix could also interfere with the biology of ECs. In other words, it affects EC’s survival, proliferation, and migration.

Decorin is a matrix proteoglycan that belongs to leucine-rich proteoglycan family [67]. It functions variously in different cellular process through binding to various tyrosine kinase receptors (RTK) and regulating the downstream signaling pathways. Decorin shows both pro-angiogenic and anti-angiogenic effects depending on molecular microenvironment. It could enhance angiogenesis through attachment to collagen I and α1β2-integrin [68]. In this process, decorin increases ECs aggregation and promotes vessel formation. On the other hand, decorin can act against angiogenesis through interacting with macromolecules including epidermal growth factor receptor (EGFR), hepatocyte growth factor (HGF), insulin-like growth factor (IGF), platelet-derived growth factor (PDGF), fibroblast growth factor (FGF). In cancer cells, decorin decreases the expression of VEGFA and HIF-1α through interfering with the signaling pathways of Hepatocyte Growth Factor (HGF/Met) and epidermal growth factor receptor (EGFR) [69]. Furthermore, decorin enhances the expression of thrombospondin-1 and provides an anti-angiogenic environment [70].

On the other hand, decorin promotes autophagosome formation, probably through interacting with VEGFR2 and enhancing paternally-expressed gene 3 (Peg3). Peg3, primarily identified as a tumor suppressor gene, encodes a transcription factor with a zinc finger domain. In ECs, Peg3 can regulate autophagy concurrent with decorin expression [67]. It could increase the expression of BECN-1/Beclin-1 and microtubule-associated protein-1 light chain-3α (LC3A) localized on autophagosomes [71, 72]. Decorin competes with VEGFA for binding to VEGFR2 and therefore decreases angiogenesis. Since VEGFR2 is specifically expressed on ECs. It is possible that the anti-angiogenic properties of decorin are exclusively exerted to the induction of autophagy in tumor tissue [73]. Furthermore, decorin can induce non-canonical autophagy through adenosine monophosphate-activated protein kinase (AMPK) induction and attenuating the anti-autophagic factors such as m-TOR and Akt located downstream of VEGFR2. Based on these data, Peg3 is an essential factor for the induction of autophagy by decorin and therefore is introduced as a new regulator of autophagy in ECs [70].

**Fig. 3:**
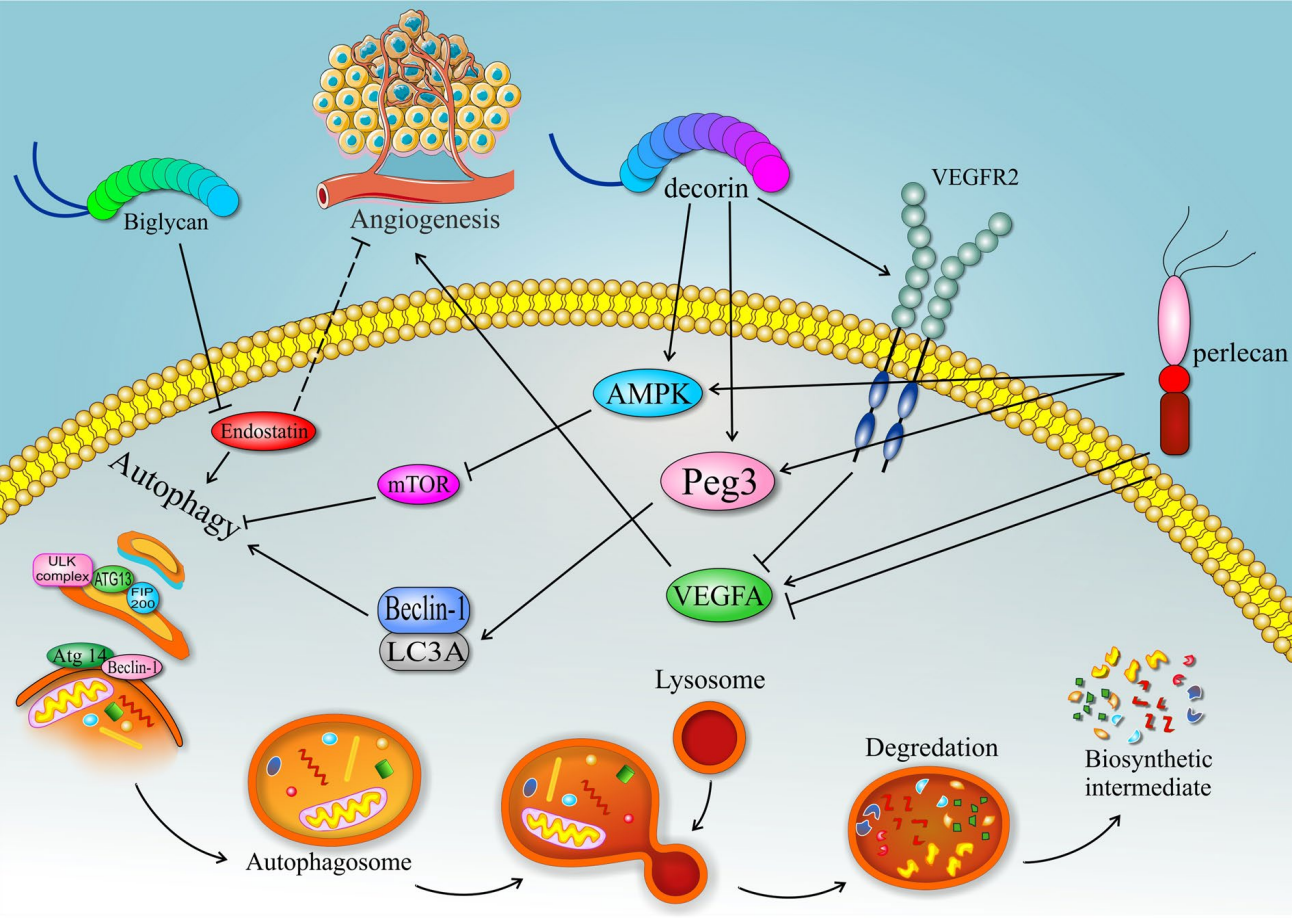
The schematic relation of autophagy and angiogenesis. Matrix proteoglycans such as Decorin, Perlecan, and Biglycancan regulate both of these pathways. Peg3 is a key molecule for angiogenesis inhibition; though it could enhance autophagy through affecting beclin-1 and LC3

The other proteoglycan associated with the membrane is Perlecan. Having several domains has rendered this molecule a multi-functional feature. The N-terminal domain of Perlecan facilitates VEGFA and fibroblast growth factor (FGF) binding to their receptors which in turn promotes angiogenesis. The C-terminal domain, however, is called Endorepellin and shows similar features as decorin [74]. Endorepellin augments anti-angiogenic environment in ECs tumor growth. In a study by Chiara Poluzziet al, it was shown that Endorepellin can inhibit angiogenesis by binding to angiostatic receptors of VEGFR2 and α2β1 integrin. On the other hand, it induces the expression of two main autophagy-related genes of *Beclin*-*1* and *LC3A*. Also, it can increase the expression of peg3 and its co-localization with *Beclin*-*1* and *LC3A* on autophagosomes [75, 76]. Since both decorin and endorepellin can inhibit tumor growth and enhance autophagy, it seems that they exert anti-angiogenic effects through autophagy pathway [70]. Endostatin and Kringle-5, a plasminogen-derived anti-angiogenesis molecule, are the endogenous inhibitors of angiogenesis that lead to a significant increase in autophagy [15]. The other molecule with the similar structure as decorin is biglycan with proangiogenic properties. Although there is no exact data on the effect of biglycan on autophagy pathway, studies show that it can inhibit endostatin. It seems that biglycan reduces autophagy through inhibiting endostatin [70]. The other pathway that connects autophagy to angiogenesis in ECs is Akt3 signaling pathway. Recent studies on mice have shown that any defect in this cascade could associate with reduced angiogenesis and elevated autophagy in ECs [77]. Accordingly, it could be interpreted that autophagy flux and angiogenesis have a negative correlation (see Fig. 3). However, this interaction is rather complicated and requires more detailed research.

### Autophagy and angiogenesis relation in cancer targeting

Targeting vessel formation and disturbing tumor vasculature have been among the primary strategies in cancer treatment. In contrary to what was initially thought, anti-angiogenesis therapy was not as successful [78]. Hypoxia induction that leads to chemotherapy resistance is the main reason behind this shortcoming. For example, the study revealed that targeting ECs in tumor microenvironment can exacerbate tumor vasculature due to hypoxia induction. This condition helps cancer cells escaping the anti-angiogenic therapy agents [79]. On the other hand, evidence shows that normalizing the tumor vasculature is more helpful for cancer treatment through improving vessel function, decreasing hypoxia, and ameliorating responses to therapy [80]. It seems that tumor vessel normalization has a close link to autophagy. Some of the cancer therapies which potentiates vessel normalization in tumor microenvironment can also affect autophagy. For example, CQ showed vascular normalization effect in addition to lysosomal inhibition. The related studies suggest that Notch1-PFKFB3 axis could be the regarding signaling pathway for exerting such effect of CQ [57, 81]. Also, the study by Li et el revealed that triptolide (TPL), as a cancer treatment agent, could inhibit angiogenesis and increase apoptosis in osteosarcoma cells. In addition, it seems that TPL can induce autophagy through Wnt/β-Catenin signaling [82]. The Wnt/β-Catenin pathway is a critical regulator of cell proliferation, angiogenesis, and cell death [83]. It has been indicated that TPL could present anti-angiogenic effects by repressing Wnt/β-Catenin signaling and boosting autophagy [82]. We discussed here our current knowledge on cancer therapy approaching both angiogenesis and autophagy processes in the context of tumor. We believe that it is important to determine the relation between autophagy and angiogenesis in tumor combinatorial therapy.

## Conclusion

Autophagy is considered a common mechanism for maintaining tumor cell survival under various stress conditions. However, the role of autophagy in tumor context is still very controversial. Recent studies have found that many compounds that target angiogenesis for cancer treatment may also affect autophagy pathway. Based on this data, the molecules and signaling pathways that link autophagy and angiogenesis together could be of great importance in cancer therapy. So, more research is required in order to find out the exact relationship between these two pathways as targeting both simultaneously may yield much more efficient results.

## Data Availability

Not applicable.

## Abbreviations

AMPK: adenosine monophosphate-activated protein kinase
Atg: autophagy-related gene
Beclin-1: Bcl-2-interacting myosin-like coiled-coil
Bif-1: Bax-binding protein-1
CMA: Chaperone-mediated autophagy
EC: endothelial cell
EGFR: epidermal growth factor receptor
FGF: fibroblast growth factor
HGF: hepatocyte growth factor
HIF: hypoxia-inducible factor
LC3A: microtubule-associated protein-1 light chain-3α
mTOR: mammalian target of rapamycin
NO: nitric oxide
Peg3: paternally-expressed gene 3
ROS: reactive oxygen species
RTKs: receptor tyrosine kinases
VEGF: vascular endothelial growth factor
